# Elements of Clinical Bacteriology for Physicians and Students

**Published:** 1900-09

**Authors:** 


ELEMENTS OF CLINICAL BACTERIOLOGY FOR PHYSICIANS AND STUDENTS. By DR. ERNST LEVY, Professor in the University of Strasburg, i. E., and DR. FELIX KLEMPERER, Privatdocent in the University of Strasburg, i. E. Second enlarged and revised edition. Authorised translation by AUGUSTUS A. ESHNER, M. D., Professor of Clinical Medicine in the Philadelphia Polyclinic, etc. Philadelphia: W. B. Saunders 1900. [Price, $2.50.]

The authors have attempted to group the results of bacteriologic investigation from the clinical point of view and, judging from the rapid exhaustion of the first edition, their efforts have been highly successful. The size of this edition is considerably increased over that of the first, owing to the results of recent investigations which have been incorporated in this one. Chapters have been added on the plague and botulism and those on immunity, diphtheria, typhoid fever, actinomycosis, examination of air and water, and others have been radically revised. The work is divided into four parts. Part I. treating of the morphology and biology of bacteria, infection, immunity and methods of culture and of examination. Part II. is devoted to inflammation and suppuration. Under this part are considered the different forms of pyogenic cocci, as the streptococci, staphylococci, pneumococci and others. The diseases occasioned by these germs are considered in this chapter as erysipelas, pleuritis, endo-and pericarditis, nephritis, abscess of the liver and the like.

Under Part III. are considered the specific diseases of bacterial origin such as typhoid fever, cholera, tuberculosis, glanders, leprosy and the like. The chapter on botulism or meat poisoning, which has been added in this second edition is a comprehensive review of this important subject. It takes up the symptoms of meat and sausage poisoning, the morphology of the bacillus botulism, as discovered by Van Ermengene, the cultural peculiarities and pathogenic properties.

Part IV. takes up the mycoses. The appendix is devoted mainly to hygiene—such as the bacteriologic examination of air, soil and water, and disinfection. Under the latter head are considered disinfecting agents—disinfection of the hands, mucous membranes, instruments and dressings, feces, cesspools, bathtubs, urine, sputum, beds, clothing, articles of food, sick-room, shops, vehicles and railway cars.

The translator has done his work admirably and has added explanatory foot-notes throughout the book. The illustrations are profuse and attractively executed. W. C. K.

